# Limited effects of the maternal rearing environment on the behaviour and fitness of an insect herbivore and its natural enemy

**DOI:** 10.1371/journal.pone.0209965

**Published:** 2019-01-11

**Authors:** Jennifer M. Slater, Lucy Gilbert, David Johnson, Alison J. Karley

**Affiliations:** 1 Institute of Biological and Environmental Sciences, University of Aberdeen, Aberdeen, United Kingdom; 2 Ecological Sciences Group, The James Hutton Institute, Invergowrie, Dundee, United Kingdom; 3 Ecological Sciences Group, The James Hutton Institute, Craigiebuckler, Aberdeen, United Kingdom; 4 School of Earth and Environmental Sciences, The University of Manchester, Manchester, United Kingdom; USDA Agricultural Research Service, UNITED STATES

## Abstract

The maternal rearing environment can affect offspring fitness or phenotype indirectly via ‘maternal effects’ and can also influence a mother’s behaviour and fecundity directly. However, it remains uncertain how the effects of the maternal rearing environment cascade through multiple trophic levels, such as in plant-insect herbivore-natural enemy interactions. Pea aphids (*Acyrthosiphon pisum*) show differential fitness on host legume species, while generalist aphid parasitoids can show variable fitness on different host aphid species, suggesting that maternal effects could operate in a plant-aphid-parasitoid system. We tested whether the maternal rearing environment affected the behaviour and fitness of aphids by rearing aphids on two plant hosts that were either the same as or different from those experienced by the mothers. A similar approach was used to test the behaviour and fitness of parasitoid wasps in response to maternal rearing environment. Here, the host environment was manipulated at the plant or plant and aphid trophic levels for parasitoid wasps. We also quantified the quality of host plants for aphids and host aphids for parasitoid wasps. In choice tests, aphids and parasitoid wasps had no preference for the plant nor plant and aphid host environment on which they were reared. Aphid offspring experienced 50.8% higher intrinsic rates of population growth, 43.4% heavier offspring and lived 14.9% longer when feeding on bean plants compared to aphids feeding on pea plants, with little effect of the maternal rearing environment. Plant tissue nitrogen concentration varied by 21.3% in response to aphid mothers’ rearing environment, and these differences correlated with offspring fitness. Maternal effects in parasitoid wasps were only observed when both the plant and aphid host environment was changed: wasp offspring were heaviest by 10.9–73.5% when both they and their mothers developed in bean-reared pea aphids. Also, parasitoid wasp fecundity was highest by 38.4% when offspring were oviposited in the maternal rearing environment. These findings indicate that maternal effects have a relatively small contribution towards the outcome of plant-aphid-parasitoid interactions.

## Introduction

The maternal rearing environment can have cascading effects on organisms and their offspring. The environment that offspring are reared in can result in preference for a particular habitat when they become adults, a process termed natal habitat preference induction [1]. For example, plants available to generalist insect herbivore species in their early life stages may also alter their knowledge of available host plants throughout the season [2], and their preference for them as oviposition sites [3]. Maternal oviposition decisions could be affected by several factors including her age and previous experience, influencing the mother’s preference for certain environments to rear their offspring which may or may not result in them selecting a more suitable environment for their offspring’s development: this has been formalised into the preference-performance or ‘mother knows best’ hypothesis [4].

Current environmental conditions can affect directly the behaviour and fecundity of organisms, while offspring phenotype and fitness can be affected by their maternal environment via ‘maternal effects’ [5]. For example, maternal effects can occur when mothers invest resources into their offspring to enhance performance using prevailing environmental conditions as cues of future environmental conditions [6,7]. Resource availability, changing abiotic conditions, host defences and abundance of predators or parasites experienced by mothers can influence their offspring phenotype. For example, field crickets (*Gryllus pennsylvanicus*) whose mothers had been exposed to a wolf spider, *Hogna helluo*, exhibited greater immobility, an anti-predatory behaviour, and higher survival rates in environments with predators than crickets produced by naïve mothers [8]. Maternal effects can also contribute to evolutionary outcomes. For example, maternal preference for a given host environment, combined with increased offspring fitness in that environment, can lead to offspring becoming adapted to maternal hosts, which could cause isolation and speciation [9]. The consequence of maternal effects is likely to be particularly complex in host-parasitoid systems, because parasitoid wasp offspring fitness could be influenced by both the host environment (e.g. different species of aphid) and the environment experienced by the host (e.g. different plant species on which aphids have been feeding).

Aphids mostly have a parthenogenetic, telescopic reproduction strategy [10], meaning that every nymph is a clonal copy of the mother and that the mother’s fitness is a key determinant of offspring fitness [11]. Due to telescopic reproduction, aphid fitness can be influenced by grand-maternal as well as maternal experience, termed transgenerational plasticity, which could lead to complex maternal effects between aphid generations [7]. Aphids may also be adapted to specific plant species, forming different ‘biotypes’, which exhibit differential fitness on leguminous host species [12,13]. However, the direct effect of mother’s fitness is often stronger than the maternal host plant environment, as the maternal plant host environment of aphids does not always have an effect on offspring survival and fecundity of aphids, as seen in milkweed-oleander aphid (*Aphis nerii*) [11] and bird cherry oat aphid (*Rhopalosiphum padi*) [14]. Conversely, the fitness of *Myzus persicae* mother aphids did not differ when feeding on chemically defended and non-defended plant hosts, but their daughters were able to anticipate a stressful environment by modifying gene expression depending on the plant host that the mother fed on [10].

Transgenerational effects in aphids and parasitoid wasps can result from maternal perception of their abiotic (e.g. low temperature, short daylength) and biotic (e.g. maternal crowding) environment. A specific component of the biotic environment is the quality of the plant and aphid species forming the parasitoid wasp maternal environment, which can alter the offspring’s oviposition choice. For example, Chesnais et al. [15] tested how changing the host plant species of black bean aphids (*Aphis fabae*) influenced oviposition by a parasitoid wasp (*Aphidius matricariae*). They showed that mother parasitoid wasps were more attracted to the plant host environment that produced offspring with the lowest fitness, but oviposition frequency was highest on the plant species that resulted in the fittest offspring [15]. Although maternal effects were not tested specifically in the latter study, they have potential to influence the regulation of aphid populations [16,17]. Parasitoid conditioning to previously-experienced host types can influence subsequent preference or willingness to oviposit [18,19], indicating that acquired and innate preferences could influence parasitoid decisions with downstream consequences for the fitness of their offspring. The mechanism underlying these effects on parasitoid wasp fitness is unknown but could be due to direct plant effects on wasp behaviour or indirect effects on aphid quality for parasitism. One way that plants could influence aphid quality for parasitism is through the provision of nutrients. Manipulation of plant quality via nitrogen fertilisation had limited effect on fitness of the parasitoid *Diaeretiella rapae* attacking two aphid species (*Myzus persicae* and *Brevicoryne brassicae*) despite significant effects on aphid fitness and aphid nutritional quality[20]. However, the effect of changing host identity at plant and aphid trophic levels on fitness of parasitoid mothers and their offspring still remains to be explored.

In this study, we investigate whether the maternal environment affects the behaviour and fitness of pea aphid (*Acyrthosiphon pisum*) and the generalist parasitoid wasp *Aphidius ervi*. Although information is limited in the literature, both generalist and specialist insects are likely to experience fitness costs when transferred between different maternal hosts, with higher costs detected for specialist aphids [21–23]. We tested attractiveness of maternal and alternative environments to adult aphids and parasitoid wasps, and quantified effects on their fecundity. Maternal effects were tested by first evaluating the impact of changing pea aphid host environment between faba bean (*Vicia faba*) and pea (*Pisum sativum*) on pea aphid offspring fitness. Second, we assessed whether changing the maternal environment of parasitoid wasps at a single trophic level, using pea aphids reared on either bean or pea, affected fitness of parasitoid wasps and their offspring. Third, we tested parasitoid wasp fitness in response to manipulation of the parasitoid wasp host environment at two trophic levels using pea aphids reared on bean or potato aphid (*Macrosiphum euphorbiae*) reared on tomato (*Solanum lycopersicum*). We hypothesised that the maternal environment would have cascading effects on the fitness of aphid and parasitoid wasp offspring. We predicted that, first, adult pea aphids and adult parasitoid wasps (Generation 0, G_0_) would prefer the plant or plant+aphid host environment that formed their own host environment (Prediction 1). Second, we predicted that pea aphid and parasitoid wasp offspring (Generation 1, G_1_) would have higher fitness on the plant or plant+aphid host environment that formed the G_0_ host environment (Prediction 2). Finally, we predicted that wasp mothers (Generation 0, G_0_) would have higher fecundity on the plant or plant+aphid host environment that formed her own developmental host environment (Prediction 3).

## Methods

### Experimental design

**Fig 1:**
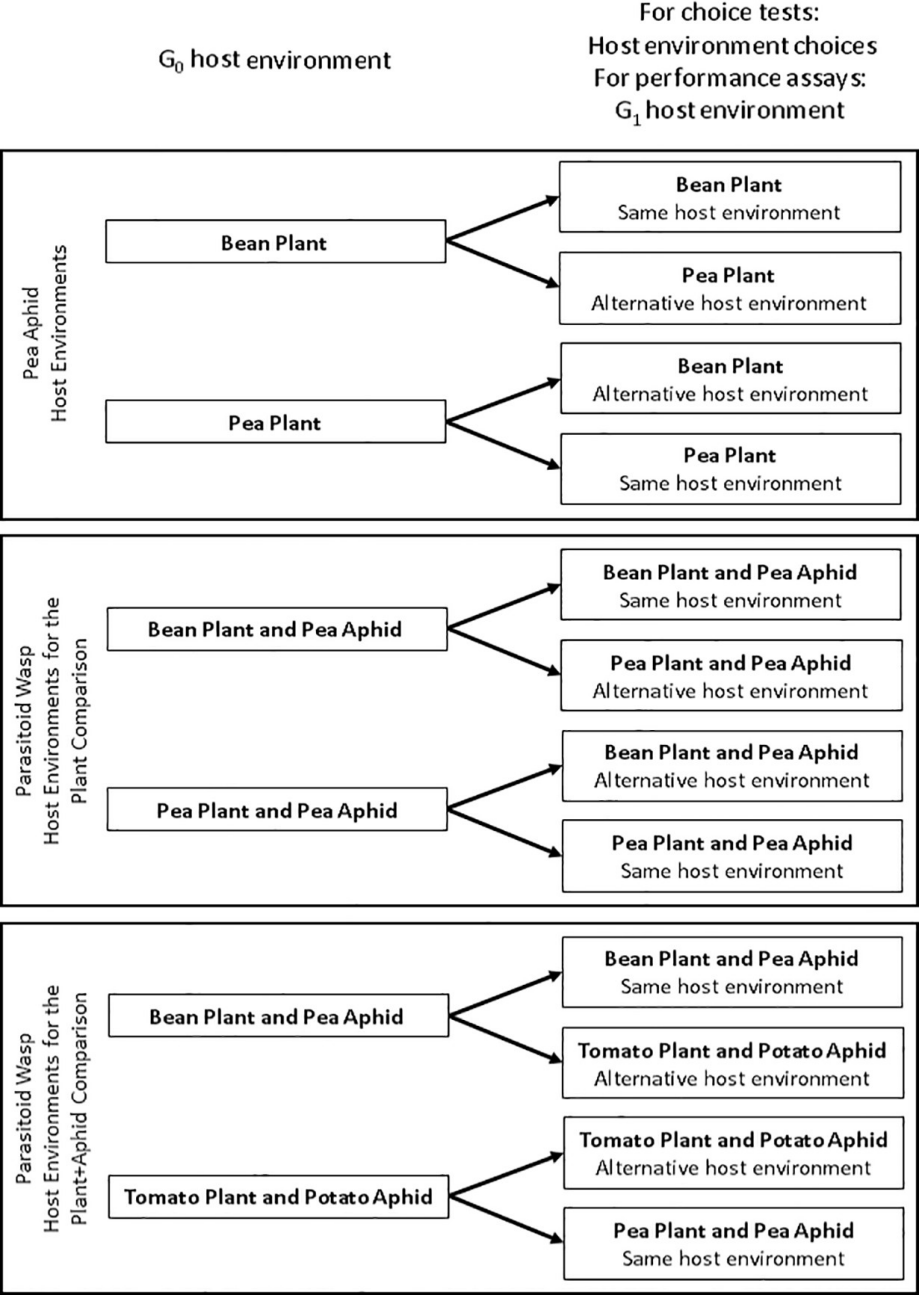
The experimental design of choice tests and performance assays. The host environment that the G_0_ insects experienced and the host environments they were offered in choice tests and that G_1_ insects were reared on in performance assays. In choice tests, adult G_0_ insects were transferred to an olfactometer connected to two host environments (see Fig 2). In performance assays, adult G_0_ insects were transferred to the same or alternative host environment immediately before nymph deposition or oviposition of G_1._ By transferring reproductive G_0_ adults, G_1_ nymphs had no prior exposure to their maternal environment.

To test the predictions, choice tests and performance assays were performed on pea aphids and parasitoid wasps. We tested the first prediction that adult G_0_ (Generation 0) pea aphids and parasitoid wasps prefer the host environment they had been reared in. Adult G_0_ insects were given a choice comprising either the host environment they had been reared in or an alternative host environment (Fig 1). Three comparisons were undertaken: i) pea aphid preferences for different plant hosts (bean *vs*. pea); and parasitoid wasp preferences for ii) pea aphids on different plant hosts (bean *vs*. pea; termed ‘plant comparison’) or iii) different plant+aphid host combinations (pea aphid on bean *vs*. potato aphid on tomato; termed ‘plant-aphid comparison’).

Performance assays were conducted for pea aphids and parasitoid wasps to test the predictions that the mother G_0_ host environment affects offspring G_1_ performance (Prediction 2) and her fecundity (Prediction 3). This assay compared G_1_ performance in the G_0_ host environment with G_1_ performance in an alternative host environment (Fig 1). The experiment comprised a cross-over design of four plant or plant+aphid host combinations. Three comparisons were undertaken: i) offspring pea aphid fitness was examined in response to two different maternal plant hosts (bean *vs*. pea) and parasitoid wasp offspring fitness was examined in response to maternal experience of ii) pea aphids on two different plant hosts (bean *vs*. pea; plant comparison) or iii) two different plant+aphid host combinations (pea aphid on bean *vs*. potato aphid on tomato; plant-aphid comparison). G_0_ pea aphids and parasitoid wasps were raised to adulthood on their designated host environment and transferred to either the same host environment or an alternative host environment and allowed to deposit G_1_ pea aphid nymphs or oviposit G_1_ parasitoid wasps. Transfer of reproductive G_0_ adults ensured that G_1_ offspring had no prior exposure to their maternal environment. For aphids, G_0_ adults were removed within 24–48 h of G_1_ nymph deposition to minimise the G_0_ mother’s effect on G_1_ offspring. We also measured the dry weight of G_2_ nymphs produced from each G_1_ adult. G_0_ mother parasitoid wasps were removed from their oviposition environment when they had oviposited in 30 aphids (see details below).

Plant and aphid host quality was assessed by quantifying tissue dry weight and nitrogen concentrations although these data could not be collected for parasitoid wasp performance assays as this would have compromised collection of mummification data.

### Plant and insect rearing

Bean (*Vicia faba* cv. Sutton Dwarf) and pea (*Pisum sativum* cv. Douce Provence) seeds were planted individually into 3L pots filled with compost (Everris, UK, 2016; base fertiliser: 156 mg/L total N, 78 mg/L P and 259 mg/L K, controlled release fertiliser: 480 mg/L total N, 117 mg/L P and 298 mg/L K and 36 mg/L Mg). Tomato (*Solanum lycopersicum* cv. Money Maker) seeds were germinated at 22°C then transferred individually into 1L pots filled with compost (as already described). Plants were grown in a greenhouse with 16:8 h (L:D) and 20°C:14°C and watered daily. Aphid clonal lines were confirmed to be free of facultative endosymbionts, such as *Hamiltonella defensa* [24], which can reduce their susceptibility to parasitism. Aphids, including G_0_, were reared on excised bean or pea cuttings (pea aphids, *Acyrthosiphon pisum*, line LL01) or excised tomato cuttings (potato aphid *Macrosiphum euphorbiae*, line AK15/01), which were replaced weekly, in ventilated plastic cups for at least four generations prior to use. This approach produced both winged and non-winged aphids in the same cup suitable for use in experiments. Winged aphid density was generally low, due to low numbers of aphids and use of high quality plant material, and these culture conditions produced a small number of winged aphids, which were sufficient in number for the olfactometer studies. Between 10–20 cups were used to culture aphids and aphids from different culture cups were mixed and randomly selected for experiments. Parasitoid wasps (*Aphidius ervi*) were obtained from Syngenta (Fargro, West Sussex, UK; batch number 31901) and all wasps used in experiments, including G_0_, were reared on pea aphids on bean or pea plants, or potato aphids on tomato plants, using aphids that were less than four days old, for at least one generation prior to experiments. When mummies had formed on plants, the leaves that the mummies had formed on were removed and placed into ventilated plastic boxes until used in experiments. Adult parasitoid wasps were fed a diluted honey solution (50% v/v) presented in cotton wool. Female parasitoid wasps, aged two to five days old and presumed mated, were used in experiments. Insect cultures were maintained at 16:8 h (L:D), 20°C:14°C and 70% humidity.

### Insect choice experiments

**Fig 2:**
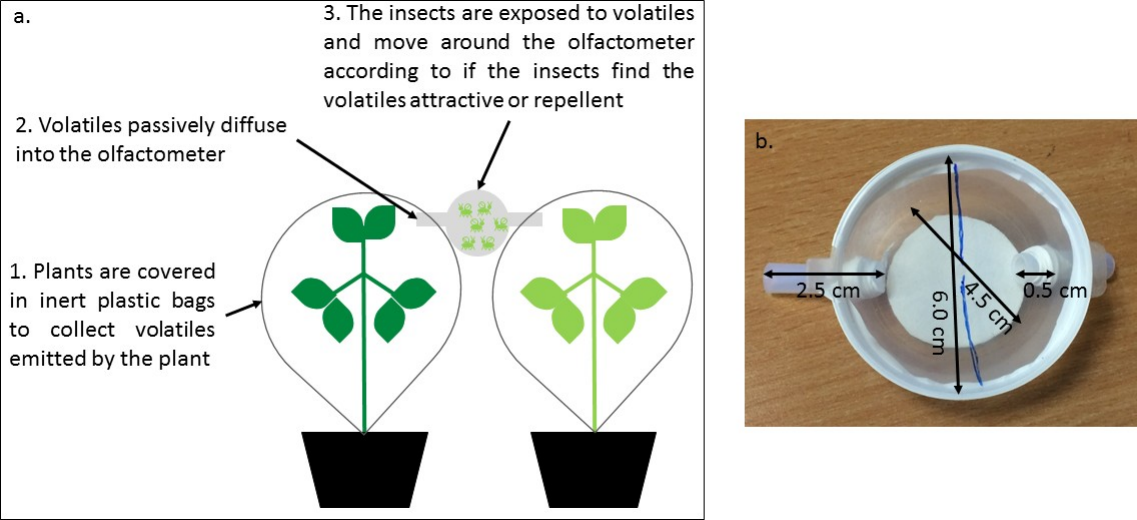
The olfactometer set up for choice tests. (A) A diagram of the experimental set-up for the choice experiments and (B) Annotated photograph of an olfactometer displaying size measurements.

Two-way choice tests were conducted with adult G_0_ winged aphids or parasitoid wasps (Fig 1). Experiments were performed under rearing conditions (see above) between 10:00 and 12:00 h using a two-armed olfactometer connected to the appropriate treatments. Three-week old plants were enclosed in polyethylene terephthalate (PET) bags and sealed at the plant base with inert plastic ties. Three days prior to parasitoid wasp choice tests, 30 aphid nymphs (2^nd^ to 3^rd^ instar) reared on the appropriate plant host were placed in mesh-covered clip cages (25 mm internal diameter) that were fixed onto leaves of experimental plants. Each arm of the olfactometer was attached to a small hole created in the corner of the PET bag enclosing experimental plants and sealed using polytetrafluoroethylene (PTFE) tape. Inert fabric, attached by PTFE tape, covered the ends of the olfactometer to prevent insect escape but allowing air flow (see Fig 2). After one hour, six winged adult pea aphids, which had been starved for a minimum of two hours, or six adult female parasitoid wasps, were placed in the centre of the olfactometer chamber and the position of each of the six insects was noted every two minutes for a 60-minute observation period. Insects were only used once. Olfactometers were cleaned for each choice test using a dilute solution of teepol, then rinsed with deionised water. Plants used in pea aphid choice tests were immediately harvested and dried at 60°C for two days to quantify total plant dry weight. A subsample of the dried leaves was randomly selected from each plant and ball milled to a fine powder. Aphids used in the parasitoid wasp choice tests were removed from plants, frozen at -20°C and freeze-dried to quantify dry weight. The tissue nitrogen concentration of milled plant material and freeze-dried aphids was determined by elemental analysis using a CE-440 Elemental Analyzer (Exeter Analytical Inc., North Chelmsford, Massachusetts, USA).

### Insect performance assays

#### Pea aphids

Two G_0_ adult pea aphids were transferred from plant cuttings to a 3-week old plant of the appropriate host environment (Fig 1) and secured by a clip cage. Experiments were conducted in glasshouse conditions (as described above for plant rearing conditions). Following production of the first cohort of G_1_ nymphs (within 24–48 h of transferring the G_0_ adult), the G_0_ aphids and all but three G_1_ nymphs were removed. The development of three G_1_ nymphs was monitored to adulthood, after which aphids were removed from the plant so that a single aphid remained in each cage. Performance of the one remaining G_1_ aphid was monitored for a maximum of twelve days of adulthood in order to calculate intrinsic rate of population growth (R_m_) [25] by:

$$Rm=0.738\times \frac{ln(Total\;number\;of\;nymphs\;(G_{2})\;produced)}{Number\;of\;days\;for\;aphid\;(G_{1})\;from\;birth\;to\;producing\;first\;G_{2}\;nymphs\;}$$

We measured the number of days the G_1_ aphid took to deposit its first nymph (development time) and the number of nymphs (G_2_) the adult G_1_ aphid produced daily (fecundity). If G_1_ pea aphids died during the experiment, the number of days the G_1_ aphid had lived was recorded as ‘survival’. Additionally, the dry weight of all G_2_ nymphs produced by each G_1_ adult was measured. Plants used in performance assays were harvested whole and dried at 60°C for two days to quantify shoot and root dry weight and leaf tissue nitrogen concentration as described above.

#### Parasitoid wasps

G_0_ wasps reared in each plant or plant+aphid host environment were transferred into a parasitism ‘arena’ comprising an excised leaf, abaxial surface uppermost, immobilised in 1% agarose (w/v) in a plastic petri dish (9 cm internal diameter). The leaf was infested with 30 aphid nymphs (2^nd^ or 3^rd^ instar); the plant or plant+aphid host environment presented in the arena reflected either the same G_0_ parasitoid wasp developmental host environment or an alternative host environment (Fig 2). Assays were performed under insect rearing conditions (described above). One mother G_0_ parasitoid wasp was introduced per arena and allowed to attack nymphs and oviposit until she had oviposited in 30 aphid nymphs; the time it took her to do this was recorded. Aphid nymphs that had been attacked were transferred onto plant cuttings of the same plant host environment used in the arena, contained in ventilated plastic cups. When an attacked nymph was removed, it was replaced with a naïve nymph to maintain a constant aphid density in the arena.

The number of mummies that developed in attacked aphids (after 14 d) was recorded as a measure of the G_0_ wasps’ fecundity. Whether or not a wasp emerged from each mummy was recorded as a measure of offspring G_1_ fitness, along with the sex and dry weight of each G_1_ wasp.

### Statistical analyses

Binomial general linear mixed models (GLMMs) were used to test for effects of host environment on pea aphid and parasitoid wasp behaviour (Prediction 1) in choice tests, using date of assay (tests were conducted over 3–7 d) and replicate as random factors. The proportion of insects (six in total, before excluding non-responders) spending time in each sector of the olfactometer chamber was calculated for each of the thirty time points for ten replicates per treatment, or eight for parasitoid wasps reared on potato aphids and tomato plants, and used in the analysis. Paired t-tests were used to test for differences in tissue dry weight and nitrogen concentration between bean and pea plant pairs used in pea aphid choice tests and for aphids used in parasitoid wasp choice tests.

For pea aphids, two-way analysis of variance (ANOVA) was used to test for maternal effects of host environment on G_1_ intrinsic rate of population growth and G_2_ nymph weight (Prediction 2). Fixed effects in the models included the G_0_ and G_1_ host environment, and the interaction, and the position of the pea aphid assay in the greenhouse was included as a random effect. Twelve replicates were used. Two-way ANOVAs were also used to test the bean and pea host dry weight and tissue nitrogen concentration with the same fixed and random effects, as described above. The correlation was tested between G_1_ intrinsic rate of population growth and host plant tissue nitrogen concentration. Pea aphid survival (measured as the number of days the G_1_ aphid lived) was analysed by fitting the survival data to a Cox proportional hazards regression model. The effect of the G_0_ and G_1_ host environment, and the interaction, was tested using analysis of deviance. During model simplification, analysis of deviance (using a Type 2 Wald chi-squared test) and Akaike’s Information Criterion (AIC) were used to check for model suitability.

Maternal effects on wasp survival (emerged vs. un-emerged wasps) and the sex (male vs. female) of the emerged G_1_ parasitoid wasps were tested using separate binomial GLMMs and logit links (due to using binary datasets) (Prediction 2). Maternal effects on G_1_ parasitoid wasp dry weight were tested using a linear mixed model (LMM). All models included the G_0_ and G_1_ host environment, and the interaction, as fixed effects and the date on which the parasitoid wasp assay was performed and the assay replicate number as a random effect. Ten replicates were used. In addition, the LMM used to analyse G_1_ parasitoid wasp dry weight included G_1_ wasp sex as a fixed factor. The ‘Anova’ function was used to obtain chi-squared, degrees of freedom and p values to establish significant differences between levels of each fixed effect. The effects of host environment on G_0_ fecundity (i.e. time taken for G_0_ parasitoid wasps to attack 30 aphid nymphs and number of wasp mummies) were analysed using two-way ANOVAs, with G_0_ and G_1_ host environment, and the interaction, as fixed effects and the date on which the wasp assay was performed as a random effect (Prediction 3). Model simplification was carried out as described above. Note that data collected for G_1_ parasitoid wasps which developed on bean-reared pea aphids, whose G_0_ parasitoid mothers also developed on bean-reared pea aphids, were used for statistical analysis in both the plant and plant-aphid comparisons.

The paired t-tests and ANOVAs were conducted using GenStat [26]. Data satisfied the requirements of parametric testing for equal homogeneity of variance and normal distribution. Survival analysis was conducted using RStudio version 3.2.4 ‘Very Secure Dishes’ [27] using the ‘coxph’ and ‘Surv’ functions in the ‘Survival’ package [28]. GLMMs and LMMs were carried out using ‘glmer’ and ‘lmer’ functions, respectively, using the ‘lme4’ [29], ‘car’ [30] and ‘lmerTest’ [31] packages in RStudio [27]. Maternal effects were indicated when the interaction between the G_0_ and G_1_ host environment was significant at the 5% level.

## Results

### Do adult (G_0_) insects prefer their rearing host environment (Prediction 1)?

#### Pea aphids

**Fig 3:**
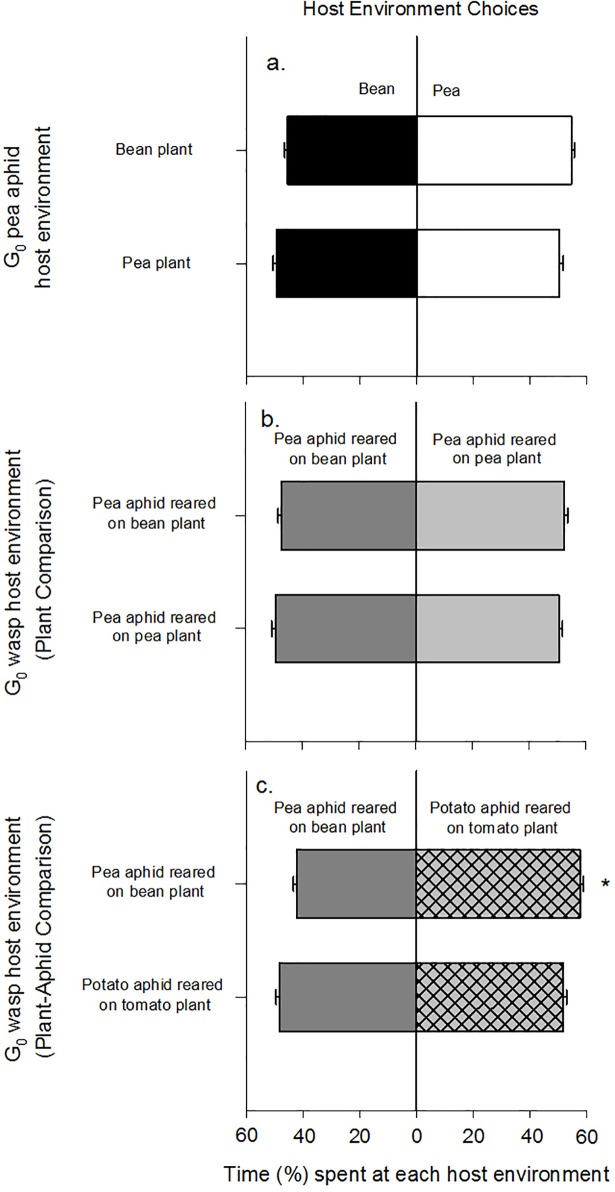
Insect host environment preferences. The percentage (%) of time spent in each half of the olfactometer chamber, exhibited by (A) pea aphids for bean (filled bars) or pea (open bars) plants, (B) parasitoid wasps for pea aphids on bean (dark grey bars) or on pea plants (light grey bars) in the plant comparison, and (C) parasitoid wasps for pea aphids on bean (dark grey bars) or potato aphids on tomato plants (light grey hatched bars) in the plant-aphid comparison. Values are means (± SEM) of n = 10 for all choice tests, except for G_0_ wasps reared on potato aphids on tomato plants when n = 8. *signifies bars are significantly different. For statistical summary see S1 Table.

G_0_ pea aphids showed no preference for bean or pea plants irrespective of the plant host environment they experienced (p>0.05; Fig 3A, S1 Table). Bean plants used in choice tests were significantly heavier than pea plants, but tissue nitrogen concentration was similar for the two species (for statistical outputs see S2 Table).

#### Parasitoid Wasps: Plant comparison

G_0_ wasps that developed in pea aphids reared on bean or pea also showed no preference for either plant type (p>0.05; Fig 3B, S1 Table). Bean-reared pea aphids used in the choice tests were significantly heavier but had the same nitrogen concentration as pea-reared pea aphids (aphid dry weight: T_18_ = 2.96, p = 0.008; aphid nitrogen concentration: T_18_ = 0.56, p = 0.585; S1 Fig).

#### Parasitoid Wasps: Plant-aphid comparison

Parasitoid wasps that had developed in bean-reared pea aphids showed a preference for tomato-reared potato aphids over bean-reared pea aphids (z = 2.445, p = 0.015; Fig 3C; S1 Table). No preference was observed for parasitoid wasps that had developed on tomato-reared potato aphids (p>0.05; Fig 3C, S1 Table). Pea aphids used in the choice tests were also heavier than potato aphids (T_17_ = 11.90, p<0.001; S1 Fig), but had significantly lower nitrogen concentration (6.21% for pea aphids *vs*. 7.21% for potato aphids, on average: T_17_ = 4.35, p<0.001; S1 Fig).

### Is insect offspring performance driven by the maternal host environment (Prediction 2)?

#### Pea aphids

**Fig 4:**
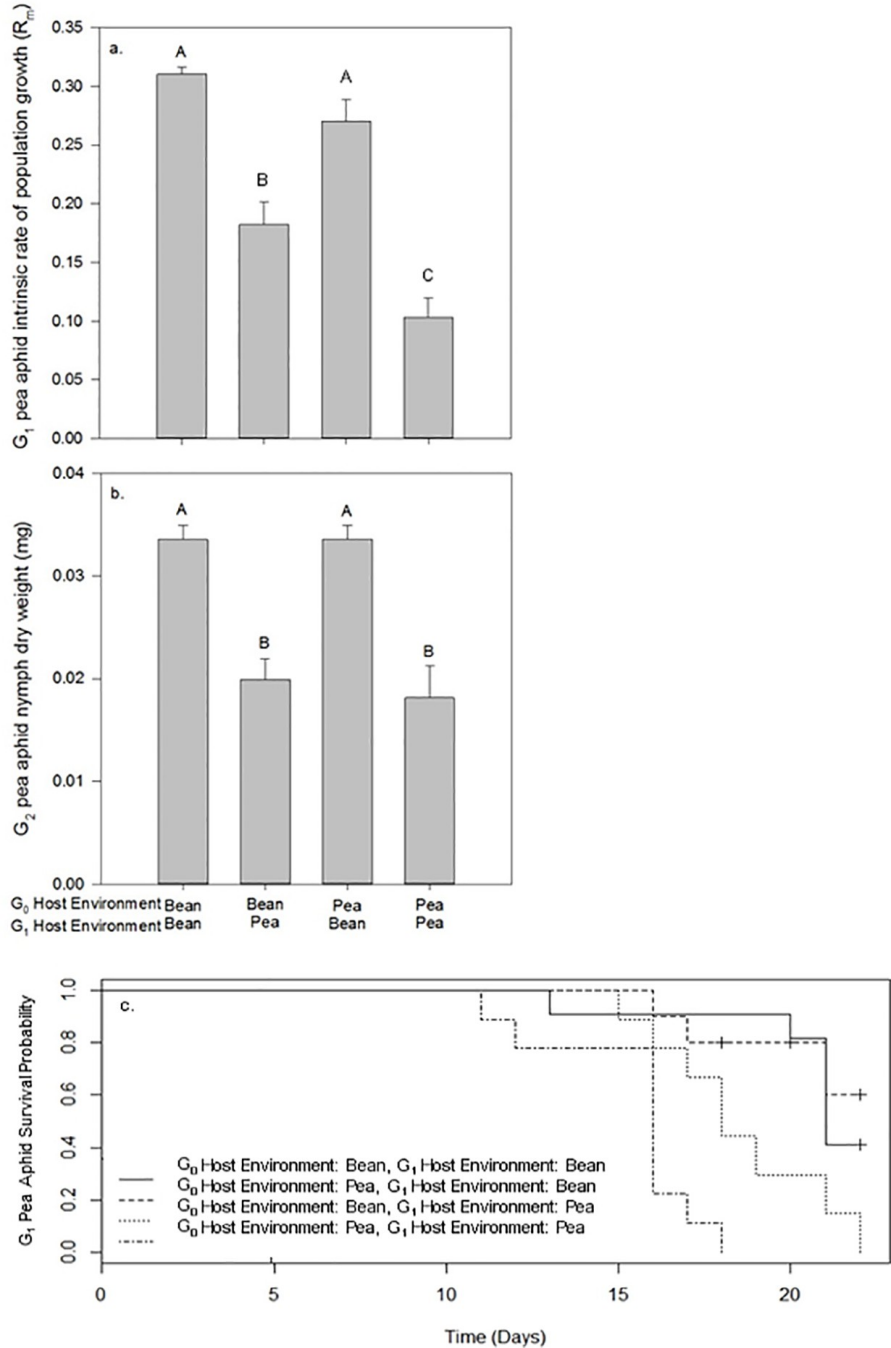
Performance of pea aphid offspring (G_1_). (A) G_1_ intrinsic rate of population increase (R_m_), (B) G_2_ nymph dry weight and (C) G_1_ survival. For (A) and (B), values are means (± SEM). Bars that share the same letter are not significantly different from each other. For (A), (B) and (C) the number of G_1_ aphid assays was n = 11 for G_0_ reared on bean and G_1_ reared on bean, n = 10 for G_0_ reared on bean and G_1_ reared on pea and G_0_ reared on pea and G_1_ reared on bean, and n = 9 for G_0_ reared on pea and G_1_ reared on pea.

**Fig 5:**
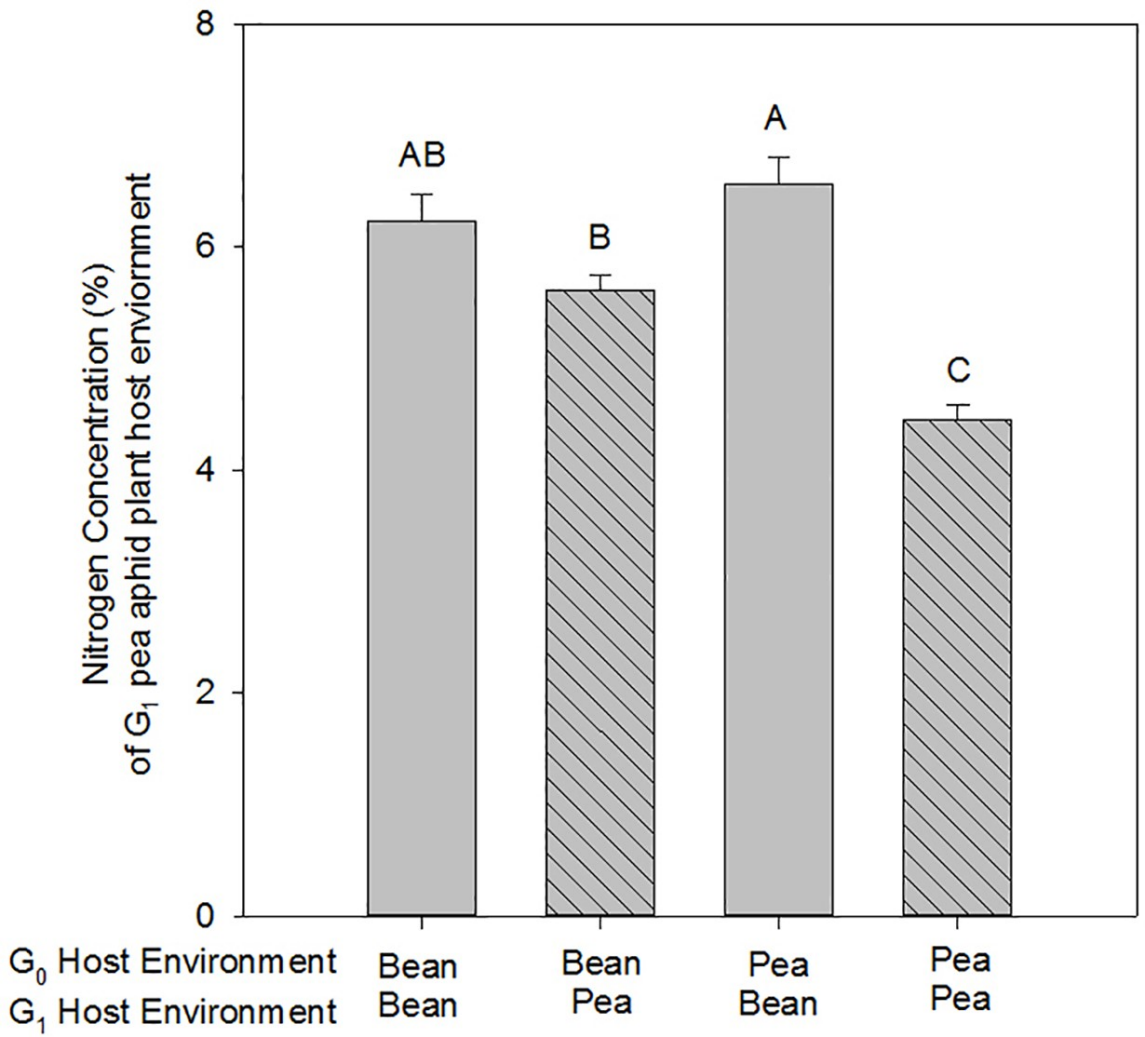
Leaf tissue nitrogen concentration of plants used in pea aphid performance assays. Bean (shaded bars) and pea (hatched bars). Values are means (± SEM) of n = 12. Bars that share the same letter are not significantly different from each other.

There was little evidence for maternal effects on G_1_ pea aphid fitness parameters. Values for G_1_ pea aphid fitness were highest when reared on bean plants: intrinsic rate of G_1_ population growth was higher (G_1_: F_1_ = 90.62, p<0.001), G_2_ nymphs were heavier (G_1_: F_1_ = 48.48, p<0.001) and G_1_ aphids survived longer (G_1_: X^2^_1_ = 21.95, p<0.001; Fig 4A–4C; S3 Table). However, when the G_0_ environment was bean, this also resulted in a higher intrinsic rate of G_1_ population growth compared to when the G_0_ environment was pea (G_0_: F_1_ = 15.47, p<0.001; Fig 4A; S3 Table), indicating a maternal effect. At the end of the performance assays, pea plants had a larger shoot dry weight (but not total weight) than bean plants, and bean plants had a higher leaf nitrogen concentration than pea plants (for statistical outputs S4 Table). There was a significant interaction between the pea aphid G_0_ and G_1_ host environment on plant tissue nitrogen concentration (G_0_*G_1_: F_1_ = 15.96, p<0.001). This was due to lower N concentrations in pea plants when infested with G_1_ pea aphids whose G_0_ mothers had been reared on pea plants (Fig 5). Indeed, the pattern observed for intrinsic rate of G_1_ population growth mirrored that of the host plant leaf tissue nitrogen concentration (Pearson’s correlation = 0.737, p<0.001).

#### Parasitoid wasps: Plant comparison

**Fig 6:**
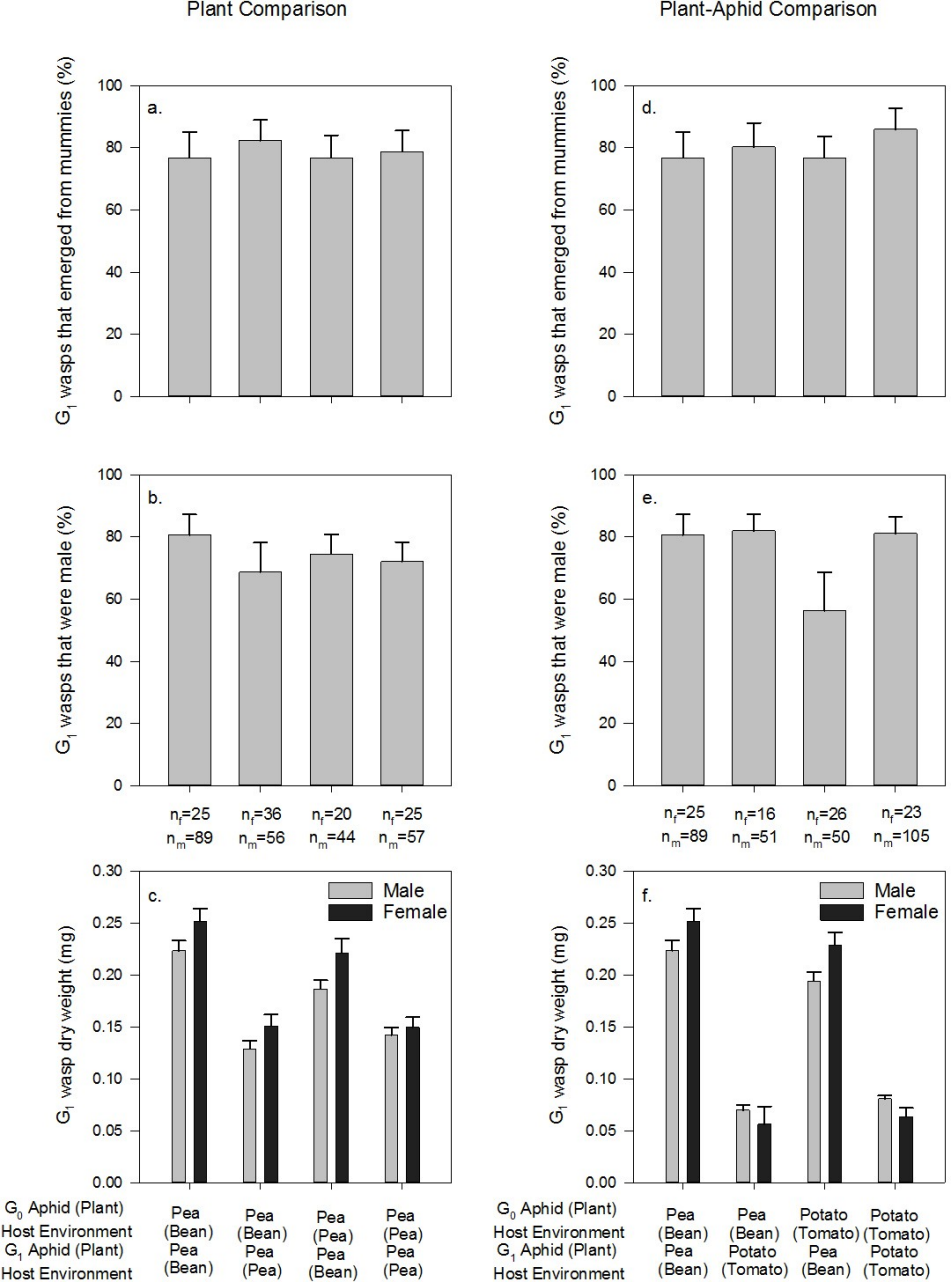
Performance of offspring (G_1_) parasitoid wasps in relation to G_0_ and G_1_ host environment. Percentage of G_1_ wasps that emerged from their mummy and survived to adulthood in (A) the Plant Comparison (p>0.05) and (D) the Plant-Aphid Comparison; the sex of G_1_ wasps in (B) the Plant Comparison and (E) the Plant-Aphid Comparison; and the weight of G_1_ wasps in (C) the Plant Comparison and (F) the Plant-Aphid Comparison. Values are means (± SEM) of n = 10 for G_0_ wasps. n_f_ and n_m_ represent the number of females and males, respectively. For statistical summaries see S5 Table.

G_1_ parasitoid wasp survival did not vary with the mother G_0_ nor offspring G_1_ host environment (p>0.05; Fig 6A; for statistical outputs see S5 Table). The majority of G_1_ emerged parasitoid wasps were male (74 ±0.2%), and G_1_ host environment had a significant effect on the sex of successfully emerged G_1_ parasitoid wasps (G_1_: X^2^_1_ = 4.817, p = 0.028; Fig 6B, S5 Table), as more males were produced from bean-reared aphids. G_1_ parasitoid wasp dry weight was not dependent on sex (p = 0.062), but the interaction between the G_0_ and G_1_ host environment was significant (G_0_*G_1_: X^2^_1_ = 8.603, p = 0.003; Fig 6C, S5 Table), with the heaviest wasps experiencing a bean-reared pea aphid G_0_ and G_1_ host environment and lightest wasps experiencing a pea-reared pea aphid G_1_ host environment.

#### Parasitoid wasps: Plant-aphid comparison

G_1_ parasitoid wasp survival to adulthood was only explained by the G_0_ host environment (G_0_: X^2^_1_ = 5.179, p = 0.023, Fig 6D, S5 Table). In accordance with the plant comparison, the majority of parasitoid wasps were male (74±0.3%), but the interaction between the G_0_ and G_1_ plant+aphid developmental host environment was significant (G_0_*G_1_: X^2^_1_ = 5.193, p = 0.023; Fig 6E, S5 Table): A smaller male sex bias was observed for wasps with a tomato-reared potato aphid G_0_ host environment and a bean-reared pea aphid G_1_ host environment compared with other host environment combinations. G_1_ parasitoid wasp weight was not explained by G_1_ parasitoid wasp sex (p = 0.480), but sex did interact with the G_1_ host environment: Females were heavier with a bean-reared pea aphid G_1_ host environment whilst males were heavier with a tomato-reared potato aphid G_1_ host environment. Also, the G_0_ and G_1_ host environment interaction was significant (G_0_*G_1_: X^2^_1_ = 13.856, p<0.001; Fig 6F, S5 Table), with the heaviest wasps experiencing a bean-reared pea aphid G_0_ and G_1_ host environment and lightest wasps experiencing a bean-reared pea aphid G_0_ and tomato-reared potato aphid G_1_ host environment.

### Is mother wasp fecundity driven by her host environment (Prediction 3)?

**Fig 7:**
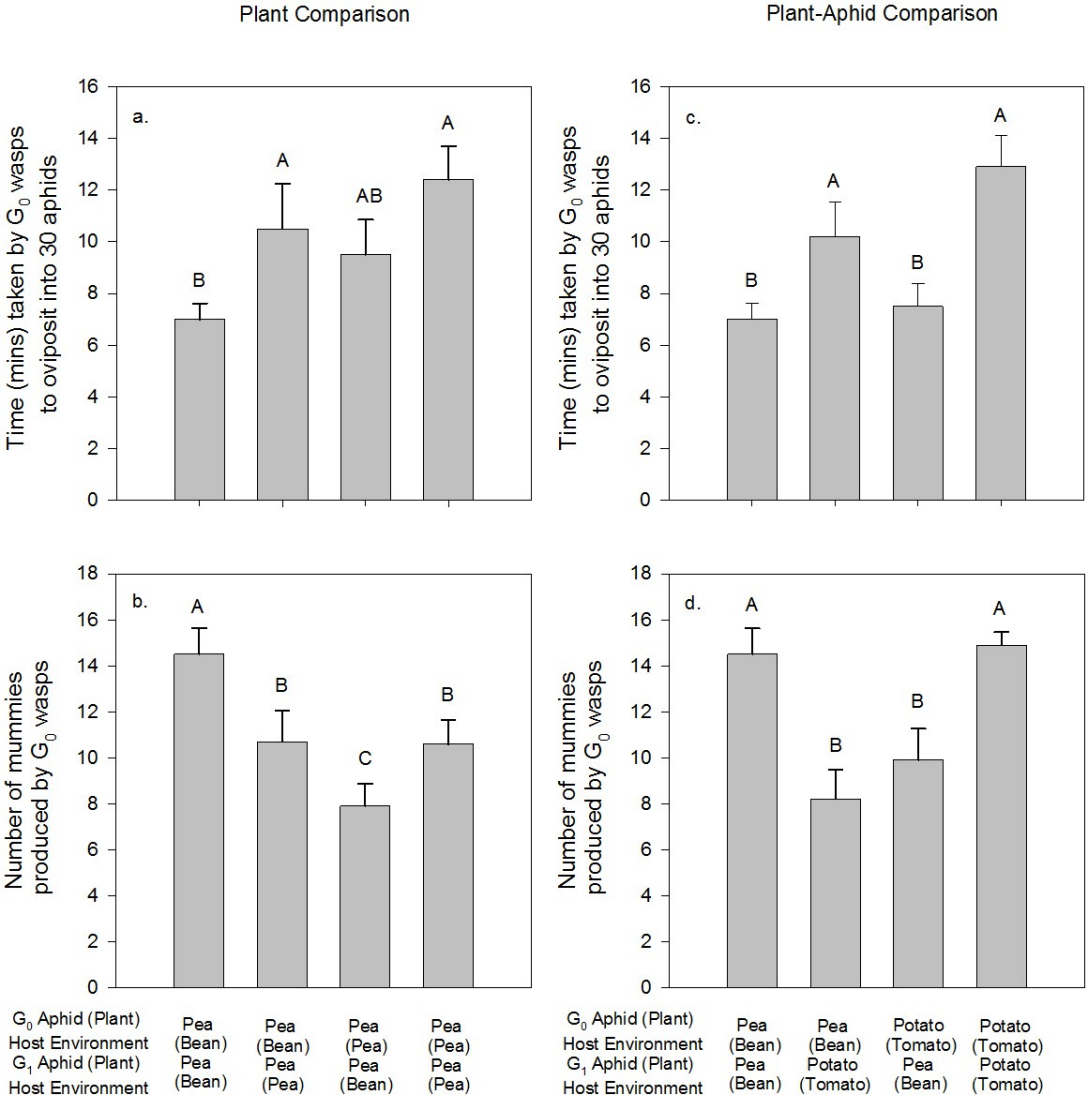
Performance of mother (G_0_) parasitoid wasps in relation to the G_0_ and G_1_ host environment. Quantified as the time (mins) taken for G_0_ wasps to oviposit into 30 aphids in (A) the Plant Comparison and (C) the Plant-Aphid Comparison and the number of G_1_ mummies formed by G_0_ wasps successfully ovipositing into aphids in (B) the Plant Comparison and (D) the Plant-Aphid Comparison. Values are means (± SEM) of n = 10. Bars that share the same letter are not significantly different from each other.

In the plant comparison, mother G_0_ parasitoid wasps attacked bean-reared pea aphid nymphs more rapidly than pea-reared pea aphid nymphs (G_1_: F_1_ = 18.01, p<0.001; Fig 7A), but the number of mummies produced per G_0_ parasitoid (and the number of subsequent G_1_ parasitoids that emerged from mummies) were highest when the maternal and offspring rearing environments were bean-reared pea aphid (G_0_*G_1_: F_1,39_ = 6.92, p = 0.014; Fig 7B). In the plant-aphid comparison, mother G_0_ parasitoid wasps attacked bean-reared pea aphids significantly faster than they attacked tomato-reared potato aphids (G_1_: F_1_ = 7.58, p = 0.010; Fig 7C), but the greatest number of mummies (and the number of subsequent G_1_ parasitoids that emerged from mummies) was observed when the G_0_ and G_1_ plant+aphid developmental host environments were the same (G_0_*G_1_: F_1,39_ = 22.07, p<0.001; Fig 7D).

## Discussion

Here we report experiments that test whether the maternal environment affects the behaviour and fitness of pea aphids and parasitoid wasps, which are both ecologically and commercially important insects. By manipulating host environments at different trophic levels, we revealed contrasting effects of maternal host environment on pea aphids and their parasitoid natural enemy, which aligned partially with our predictions.

We predicted that, given a choice, pea aphids and parasitoid wasps would preferentially select the host environment that they experienced during development. However, in all but one case, aphids and wasps showed no preference for either maternal or alternative host environments, and this finding did not support our first prediction. Only parasitoid wasps that had developed on bean-reared pea aphids showed any preferences, which was for tomato-reared potato aphids. However, the fecundity of parasitoid mothers and fitness of their offspring (assessed by their weight) were unaffected in this particular host environment.

We had expected pea aphids to prefer the host plant they were reared on, as previous studies found that pea aphid genotypes specialised to different legume plants preferred the legume they were adapted to rather than other leguminous species [12]. The olfactometer methodology we used relied on insects responding to the volatile organic compounds released by plants (see [32]), and prevented pea aphids from probing potentially favourable parts of the plant to assess host quality, including phloem sap quality [33], which ultimately affects aphid feeding. Aphids used in choice tests were reared on plant cuttings, rather than whole plants, which may have exposed the aphids to a variety of plant volatile cues and hence influenced aphid behaviour towards volatiles in the olfactometer tests. The leaf tissue nitrogen and carbon concentrations and C:N ratio (data not shown) were similar between bean and pea plants used in choice tests, which may have contributed to the lack of pea aphid preference, although recent work has also found that leaf phosphorus concentrations do not drive aphid attractiveness to bean plants [34].

Like aphids, parasitoid wasps use a variety of methods to assess host quality in order to make the ultimate decision to oviposit using a variety of visual and physical cues. These include aphid size (instar), shape, colour and movement [35,36], probing aphid hosts and aphid defensive behaviours, like body raising, kicking and body rotation [37]. Wasps were unable to use these cues to test for host quality in our choice tests and were only able to use plant volatiles as cues. We found that wasps reared on bean-reared pea aphids preferred tomato-reared potato aphids, suggesting preference for volatiles released by tomato plants. *Aphidius ervi* has been shown to respond to similar volatiles, including methyl salicylate, released from both pea aphid-infested bean plants [38] and potato aphid-infested tomato plants[39], and we therefore need to undertake more detailed analyses of volatile compounds released from host plant leaves to provide a mechanistic understanding of our findings.

The nitrogen concentration of pea aphids reared on either bean or pea plants were similar, but potato aphids had higher nitrogen concentration than pea aphids in choice tests. However, pea aphids were bigger than potato aphids, and hence did not offer a larger nitrogen resource overall. Plant nitrogen status has been shown to influence volatile emissions: in soybean plants (*Glycine max*), nitrogen-starved plants had a similar volatile composition to that of nitrogen-fertilised plants, but three compounds were released in different quantities; however, these differences were undetectable when plants were attacked by fall armyworms (*Spodoptera frugiperda*) and parasitoid wasps (*Cotesia marginiventris*) showed no preference between infested plants that had been nitrogen starved or fertilised [40]. The olfactory cues influencing parasitoid wasp choice in our study remain to be elucidated, particularly how they might relate to plant and aphid nutrient status.

The performance of aphid offspring, measured by nymph weight and survival, experiencing the same host environment as their mother’s host environment did not differ from those experiencing an alternative plant host environment. However, the intrinsic rate of population increase was explained by the maternal and offspring environment (although not by an interaction), lending a little support for our second prediction. Pea aphid offspring had a higher intrinsic rate of population increase, heavier nymphs and survived longer on a bean host environment. Maternal environment might be anticipated to be a key determinant of offspring fitness in clonally-reproducing aphids. Previous research has shown, however, that pea aphids perform as well or better on bean plants regardless of their maternal hosts [13] and McLean et al. [7] also found no maternal effects on offspring fecundity of host-adapted pea aphid genotypes across multiple generations when host swapping between *Lathyrus pratensis* and bean plants.

Aphid infestation [41] or infestation by different aphid species [42,43] often leads to differences in plant tissue nutrient concentrations, including nitrogen and soluble amino acids. Here, we report that the prior plant host environment of an individual aphid can affect the nutrient concentration of their host plant and that this is positively correlated with aphid intrinsic rate of population growth. Lower leaf tissue nitrogen concentrations and aphid intrinsic rate of population growth were observed in pea plants harbouring pea aphids whose mothers had fed on pea rather than bean plants. Aphids tend to be nitrogen limited [44] and these findings indicate that maternal plant quality can affect aphid physiology, feeding and fitness in a way that influences the aphid’s ability to utilise future plant host resources. Further work might reveal the mechanism of this effect, for example via induced resource sequestration [45], where resources are re-allocated to different plant structures. Indeed, measuring nitrogen content of phloem sap may shed further light on this phenomenon.

Our second prediction, that parasitoid wasp offspring fitness proxies would be highest when offspring experienced their mother’s environment, was not supported by offspring wasp emergence and wasp sex ratio data when only maternal plant environment was manipulated (i.e. plant comparison). However, in the plant-aphid comparison, parasitoid offspring had a lower male bias with a tomato-reared potato aphid maternal host environment and bean-reared pea aphid offspring host environment compared to other host environment combinations. *A*. *ervi* has a haploid-diploid reproduction strategy [46], allowing mothers to choose whether to oviposit sons or daughters into aphid hosts. This choice is often decided by assessment of host quality, which could include the lipid content of aphid hosts [47]. In dense populations mothers choose to produce more males to outcompete mating rivals for females [46, 48]; mass rearing in the laboratory can simulate these conditions [48], which could explain why the majority of wasps observed in our study were male. Alternatively, mother wasps, although presumed mated based on observations in the wasp cultures, might not have been mated prior to experiments. However, mothers with a developmental host environment of tomato-reared potato aphids oviposited more daughters in bigger pea aphids that represent a more plentiful resource for offspring. Offspring wasp body mass is primarily determined by aphid body mass [49], but in our study the offspring host environment also interacted with the maternal host environment in both the plant and plant-aphid comparisons. Specifically, the biggest wasps were those that developed in pea aphids reared on bean in both the maternal and offspring generations. This finding demonstrates that bean reared pea aphids are an overall better host, but these beneficial effects are maximised when mother wasps also developed in bean-reared pea aphids: this supports our second prediction and evidences potential maternal effects. Body mass is an important fitness indicator for parasitoid wasps because it is often positively correlated with longevity, host and mate searching rate, fecundity and ability to parasitize [50]. The benefits of producing larger and fitter offspring could explain why G_0_ wasps oviposited more rapidly in the largest aphid hosts (bean-reared pea aphids) in both the plant and plant-aphid comparisons, but this preference was not observed in choice tests. However, smaller wasps were produced in a different plant+aphid host environment compared to only a different plant host environment: Offering wasps an aphid host that represented a resource of differing quality seemed to have a stronger impact on wasp fitness than changing the plant aspect of the host environment, although these factors are linked, making interpretation complex.

These findings can help inform biological control methods. For example, by choice of a favourable host environment combination, mass rearing of parasitoid wasps could be optimised to maximise parasitoid wasp mothers’ fecundity and improve the potential of their offspring to regulate aphid pests. However, the fitness of the offspring is likely to be maximised using larger aphid hosts, which could result in mother-offspring conflict if mothers preferentially oviposit in smaller aphids, resulting in short-term negative maternal effects on offspring fitness that compromise long-term aphid biocontrol. Further work is needed to understand the impact of environmental heterogeneity and trophic complexity on maternal effects. For example, positive maternal effects on offspring fitness are likely to be compromised by fluctuations in the insect environment that lead to unpredictability in host quality and suitability. An interesting avenue for further work is to determine whether the positive maternal effects observed in this study could affect other trophic levels (e.g. hyperparasitoids) and lead to cascading maternal effects through the food web. The hypotheses tested in this study could be applied to additional trophic groups, although challenging to test empirically due to the experiment size doubling each time a treatment level is added.

Our study shows that the plant and aphid host environments of wasps can interact to affect wasp fitness, but the exact outcomes are complex and inconsistent. Specifically, wasps reared on bean plants and pea aphids appeared to have the highest fitness, possibly as pea aphids themselves had the highest fitness on bean plants. In order to better gather evidence for maternal effects in wasps, more plant and aphid host environment combinations need to be tested. This information could help facilitate the most appropriate rearing environments used in the biocontrol industry.

## Supporting information

S1 Fig Quality of aphids used in G_0_ wasp choice tests. Aphid dry weight (mg) for (A) the Plant Comparison and (C) the Plant-Aphid Comparison. Aphid nitrogen concentration for (B) the Plant Comparison and (D) the Plant-Aphid Comparison. Values are means (± SEM) of n = 20 for pea aphids reared on bean plants and n = 19 pea aphids reared on pea plants in the Plant Comparison and n = 18 for pea aphids reared on bean plants and n = 18 for potato aphids reared on tomato plants for the Plant-Aphid Comparison. ** p<0.01, *** p<0.001.
(TIF)

S1 Table Statistical summaries of the pea aphid and wasp choice tests shown in Fig 3. Negative estimates and z values indicate preference for the ‘alternative’ compared to the ‘same’ host environment (Fig 1) presented in the choice tests. Significant preferences are highlighted in bold.
(DOCX)

S2 Table Details on plants used in G_0_ pea aphid choice tests. Weight (g) and leaf nitrogen concentration (% dry mass) of 3-week old bean. Values are means (± SEM) of n = 12 plants. Significant differences are highlighted in bold.
(DOCX)

S3 Table Statistical summaries of two-way ANOVAs for pea aphid performance. G_1_ intrinsic rate of population increase (R_m_), G_2_ nymph dry weight and G_1_ survival for pea aphids used in performance assays (Fig 4). Significant differences are highlighted in bold.
(DOCX)

S4 Table Details on plants used in G_1_ pea aphid performance assays. Weight (g) and leaf nitrogen concentration (% dry mass) of six-week old bean and pea plants, after being infested with G_1_ pea aphids for three weeks. Significant differences are highlighted in bold.
(DOCX)

S5 Table Summary statistics of (general) linear mixed models ((G)LMMs) for wasp performance. GLMMS for G_1_ wasp survival and sex, and LMMs for G_1_ wasp weight were used to test for maternal effects for the Plant and Plant-Aphid Comparison (Fig 6). Statistical outputs are provided from the most simplified models (see text for details). Significant results are highlighted in bold.
(DOCX)
