# Supplementary figures and images for: Limited effects of the maternal rearing environment on the behaviour and fitness of an insect herbivore and its natural enemy
Source: PLoS One. 2019 Jan 11;14(1):e0209965. doi: 10.1371/journal.pone.0209965 (PMC6329576; doi:10.1371/journal.pone.0209965)

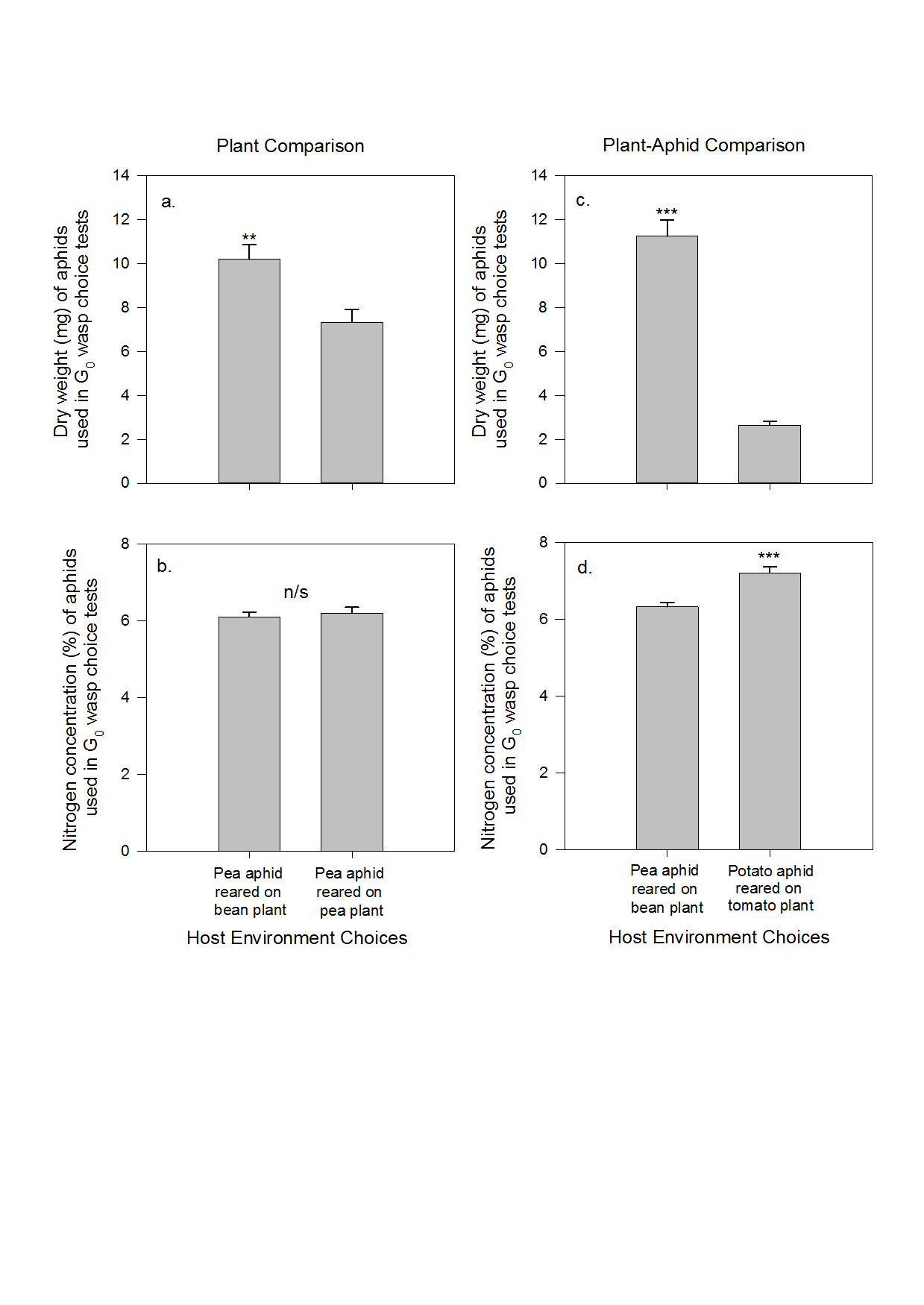

Supplement: S1 Fig — Aphid dry weight (mg) for (A) the Plant Comparison and (C) the Plant-Aphid Comparison. Aphid nitrogen concentration for (B) the Plant Comparison and (D) the Plant-Aphid Comparison. Values are means (± SEM) of n = 20 for pea aphids reared on bean plants and n = 19 pea aphids reared on pea plants in the Plant Comparison and n = 18 for pea aphids reared on bean plants and n = 18 for potato aphids reared on tomato plants for the Plant-Aphid Comparison. ** p<0.01, *** p<0.001. (TIF) [file pone.0209965.s001.tif]
