# Supplementary material for: Limited effects of the maternal rearing environment on the behaviour and fitness of an insect herbivore and its natural enemy
Source: PLoS One. 2019 Jan 11;14(1):e0209965. doi: 10.1371/journal.pone.0209965 (PMC6329576; doi:10.1371/journal.pone.0209965)
Supplement: S1 Table — Negative estimates and z values indicate preference for the ‘alternative’ compared to the ‘same’ host environment (Fig 1) presented in the choice tests. Significant preferences are highlighted in bold. (DOCX) [file pone.0209965.s002.docx]

**Supporting Table 1. Statistical summaries of the pea aphid and wasp choice tests shown in Figure 3.** Negative estimates and z values indicate preference for the ‘alternative’ compared to the ‘same’ host environment (Figure 1) presented in the choice tests. Significant preferences are highlighted in bold.

| Experiment | G_0_ Host Environment | *N* | Estimate | Standard Error | *Z* | *P* |
| --- | --- | --- | --- | --- | --- | --- |
| Pea Aphids | Bean Plant | 10 | -0.188 | 0.140 | -1.344 | 0.179 |
|  | Pea Plant | 10 | 0.159 | 0.198 | 0.803 | 0.422 |
| Wasps  (Plant Comparison) | Bean Plant and Pea Aphids | 10 | -0.101 | 0.073 | -1.386 | 0.166 |
|  | Pea Plant and Pea Aphids | 10 | 0.080 | 0.104 | 0.775 | 0.438 |
| Wasps  (Plant-Aphid Comparison) | Bean Plant and Pea Aphids | 10 | -0.318 | 0.130 | -2.445 | **0.015** |
|  | Tomato Plant and Potato Aphids | 8 | 0.327 | 0.195 | 1.677 | 0.096 |
