# Supplementary material for: Limited effects of the maternal rearing environment on the behaviour and fitness of an insect herbivore and its natural enemy
Source: PLoS One. 2019 Jan 11;14(1):e0209965. doi: 10.1371/journal.pone.0209965 (PMC6329576; doi:10.1371/journal.pone.0209965)
Supplement: S2 Table — Weight (g) and leaf nitrogen concentration (% dry mass) of 3-week old bean. Values are means (± SEM) of n = 12 plants. Significant differences are highlighted in bold. (DOCX) [file pone.0209965.s003.docx]

**Supporting Table 2. Details on plants used in G_0_ pea aphid choice tests.** Including weight (g) and leaf nitrogen concentration (% dry mass) of 3-week old bean. Values are means (± SEM) of n=12 plants. Significant differences are highlighted in bold.

| Variable | Bean (± 1 S.E.) | Pea (± 1 S.E.) | *T* | df | *P* |
| --- | --- | --- | --- | --- | --- |
| Shoot biomass (g) | 0.607 (± 0.0381) | 0.453 (± 0.0169) | 3.76 | 23 | **0.001** |
| Shoot + root biomass (g) | 1.278 (± 0.0595) | 0.676 (± 0.0270) | 9.95 | 23 | **<0.001** |
| Leaf nitrogen concentration (%) | 7.87 (± 0.248) | 7.85 (± 0.210) | 0.07 | 23 | 0.944 |
