# Supplementary material for: Limited effects of the maternal rearing environment on the behaviour and fitness of an insect herbivore and its natural enemy
Source: PLoS One. 2019 Jan 11;14(1):e0209965. doi: 10.1371/journal.pone.0209965 (PMC6329576; doi:10.1371/journal.pone.0209965)
Supplement: S3 Table — G1 intrinsic rate of population increase (Rm), G2 nymph dry weight and G1 survival for pea aphids used in performance assays (Fig 4). Significant differences are highlighted in bold. (DOCX) [file pone.0209965.s004.docx]

**Supporting Table 3. Statistical summaries of two-way ANOVAs for pea aphid performance.** G_1_ intrinsic rate of population increase (R_m_), G_2_ nymph dry weight and G_1_ survival were used to test for maternal effects for the pea aphid performance assays (Figure 4). Significant differences are highlighted in bold.

|  |  | G_1_ R_m_ | | G_2_ Dry Weight (mg) | | G_1_ Survival Probability | |
| --- | --- | --- | --- | --- | --- | --- | --- |
|  | df | *F* | *P* | *F* | *P* | *X^2^* | *P* |
| G_0_ | 1 | 15.47 | **<0.001** | 2.251 | 0.142 | 0.60 | 0.439 |
| G_1_ | 1 | 90.62 | **<0.001** | 48.48 | **<0.001** | 21.95 | **<0.001** |
| G_0_*G_1_ | 1 | 1.57 | 0.219 | 0.027 | 0.871 | 2.28 | 0.131 |
