# Supplementary material for: Limited effects of the maternal rearing environment on the behaviour and fitness of an insect herbivore and its natural enemy
Source: PLoS One. 2019 Jan 11;14(1):e0209965. doi: 10.1371/journal.pone.0209965 (PMC6329576; doi:10.1371/journal.pone.0209965)
Supplement: S4 Table — Weight (g) and leaf nitrogen concentration (% dry mass) of six-week old bean and pea plants, after being infested with G1 pea aphids for three weeks. Significant differences are highlighted in bold. (DOCX) [file pone.0209965.s005.docx]

**Supporting Table 4. Details on plants used in G_1_ pea aphid performance assays.** Including weight (g) and leaf nitrogen concentration (% dry mass) of six-week old bean and pea plants, after being infested with G_1_ pea aphids for three weeks. Significant differences are highlighted in bold.

|  |  | Shoot biomass (g) | | Shoot + root biomass (g) | | Leaf nitrogen concentration (%) | |
| --- | --- | --- | --- | --- | --- | --- | --- |
|  | df | *F* | *P* | *F* | *P* | *F* | *P* |
| Block | 3 | 0.67 |  | 0.69 |  | 1.51 |  |
| G_0_ | 1 | 0.01 | 0.919 | 0.42 | 0.519 | 4.74 | **0.035** |
| G_1_ | 1 | 11.26 | **0.002** | 0.15 | 0.705 | 51.93 | **<0.001** |
| G_0_*G_1_ | 1 | 0.66 | 0.422 | 0.43 | 0.515 | 15.96 | **<0.001** |
| Error | 47 |  |  |  |  |  |  |
