# Supplementary material for: Limited effects of the maternal rearing environment on the behaviour and fitness of an insect herbivore and its natural enemy
Source: PLoS One. 2019 Jan 11;14(1):e0209965. doi: 10.1371/journal.pone.0209965 (PMC6329576; doi:10.1371/journal.pone.0209965)
Supplement: S5 Table — GLMMS for G1 wasp survival and sex, and LMMs for G1 wasp weight were used to test for maternal effects for the Plant and Plant-Aphid Comparison (Fig 6). Statistical outputs are provided from the most simplified models (see text for details). Significant results are highlighted in bold. (DOCX) [file pone.0209965.s006.docx]

**Supporting Table 5. Summary statistics of general linear mixed models (GLMMs) for wasp performance.** G_1_ wasp survival and sex, and linear mixed models (LMMs) for G_1_ wasp weight were used to test for maternal effects for the Plant and Plant-Aphid Comparison (Figure 6). Statistical outputs are provided from the most simplified models (see text for details). Significant results are highlighted in bold.

| Comparison | Variable | Fixed Effects | *Χ^2^* | df | *P* |
| --- | --- | --- | --- | --- | --- |
| Plant | Survival | G_0_ Host Environment | 1.77 | 1 | 0.184 |
|  |  | G_1_ Host Environment | 1.06 | 1 | 0.304 |
|  |  | G_0_ Host Environment * G_1_ Host Environment | 1.75 | 1 | 0.186 |
|  | Sex | G_1_ Host Environment | 4.82 | 1 | **0.028** |
|  | Weight | Sex | 3.48 | 1 | 0.062 |
|  |  | G_0_ Host Environment | 1.19 | 1 | 0.275 |
|  |  | G_1_ Host Environment | 138.4 | 1 | **<0.001** |
|  |  | G_0_ Host Environment * G_1_ Host Environment | 8.60 | 1 | **0.003** |
| Plant-Aphid | Survival | G_0_ Host Environment | 5.18 | 1 | **0.023** |
|  | Sex | G_0_ Host Environment | 1.11 | 1 | 0.292 |
|  |  | G_1_ Host Environment | 2.56 | 1 | 0.110 |
|  |  | G_0_ Host Environment * G_1_ Host Environment | 5.19 | 1 | **0.023** |
|  | Weight | Sex | 0.50 | 1 | 0.480 |
|  |  | G_0_ Host Environment | 0.80 | 1 | 0.372 |
|  |  | G_1_ Host Environment | 552.6 | 1 | **<0.001** |
|  |  | Sex * G_1_ Host Environment | 8.68 | 1 | **0.003** |
|  |  | G_0_ Host Environment * G_1_ Host Environment | 13.86 | 1 | **<0.001** |
